# Race and Regional Differences in Risk Factors for Cognitive Impairment From a Combined Cohort Analysis

**DOI:** 10.1093/geroni/igaa057.2828

**Published:** 2020-12-16

**Authors:** B Gwen Windham, Michael Griswold, James Henegan, David Knopman, Seth Lirette, Michelle Mielke, Ron Petersen, Thomas Mosley

**Affiliations:** 1 University of Mississippi Medical Center, Jackson, Mississippi, United States; 2 The University of Mississippi Medical Center, Jackson, Mississippi, United States; 3 Mayo Clinic, Rochester, Minnesota, United States

## Abstract

Few studies examined race-regional influences on mild cognitive impairment (MCI) and dementia risk factors. We examined 14 risk factors in 5 race-regions, e.g. cross-sectional age- and sex-adjusted relative prevalence ratios (RPR) from multinomial regression comparing associations of low education (<12yrs) with MCI and dementia (versus normal cognition) across 3 race-region groups, average age 76yrs: whites in the Mayo Clinic Study of Aging[MCSA, Olmsted County, MN—MCSA-MN-Whites); Atherosclerosis Risk in Communities(ARIC) whites in suburban Minneapolis, MN—ARIC-MN-Whites); ARIC-African Americans in Jackson, MS—ARIC-MS-AA. Among 3,787 MCSA-Whites, low education exerted a 2-fold risk for MCI, RPR=2.09(95%CI: 1.57,2.78). Conversely, low education was not a supported MCI risk factor for ARIC-MN-Whites (n=1,901—RPR=0.63(0.31,1.28) or ARIC-MS-AA, (n=1,416—RPR=.0.81 (0.60,1.10)), with substantially differential race-region effects. Low education RPRs for dementia also differed by race-region: RPR=4.43(2.68,7.31-MCSA-MN-W), RPR=0.70 (0.16,2.99-ARIC-MN-Whites), and RPR=2.54 (1.74,3.72-ARIC-MS-AA). Understanding why risk factors differ by race and region likely requires diverse samples and harmonized methods using culturally-appropriate assessments.

